# Cadmium and Its Neurotoxic Effects

**DOI:** 10.1155/2013/898034

**Published:** 2013-08-12

**Authors:** Bo Wang, Yanli Du

**Affiliations:** ^1^Department of Pathology, The Second Clinical Medical School of Inner Mongolia University for the Nationalities (Inner Mongolia Forestry General Hospital), Yakeshi 022150, Inner Mongolia, China; ^2^Department of Neurosurgery, The Second Clinical Medical School of Inner Mongolia University for the Nationalities (Inner Mongolia Forestry General Hospital), Yakeshi 022150, Inner Mongolia, China

## Abstract

Cadmium (Cd) is a heavy metal that has received considerable concern environmentally and occupationally. Cd has a long biological half-life mainly due to its low rate of excretion from the body. Thus, prolonged exposure to Cd will cause toxic effect due to its accumulation over time in a variety of tissues, including kidneys, liver, central nervous system (CNS), and peripheral neuronal systems. Cd can be uptaken from the nasal mucosa or olfactory pathways into the peripheral and central neurons; for the latter, Cd can increase the blood brain barrier (BBB) permeability. However, mechanisms underlying Cd neurotoxicity remain not completely understood. Effect of Cd neurotransmitter, oxidative damage, interaction with other metals such as cobalt and zinc, estrogen-like, effect and epigenetic modification may all be the underlying mechanisms. Here, we review the *in vitro* and *in vivo* evidence of neurotoxic effects of Cd. The available finding indicates the neurotoxic effects of Cd that was associated with both biochemical changes of the cell and functional changes of central nervous system, suggesting that neurotoxic effects may play a role in the systemic toxic effects of the exposure to Cd, particularly the long-term exposure.

## 1. Introduction

**Figure 1:**
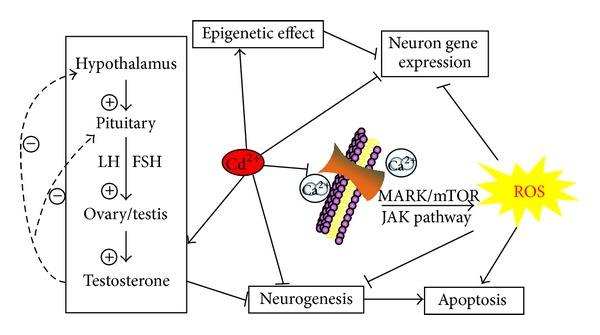
Mechanistic illustration of the neuronal toxicity of Cd. (1) Cd-induced neuron cell apoptosis and ROS (reactive oxygen species) are mediated through Ca^2+^-mitochondria signaling and Ca^2+^-membrane channels. (2) Cd impaired neurogenesis. (3) Cd accumulation in the brain leads to the altered gene expression and epigenetic effect. (4) Cd has estrogen-like effect, which can induce endocrine disruption by affecting the hypothalamic-pituitary-gonadal (HPG) axis in different aspects. Meanwhile, these potential mechanisms have possible interactions. The solid black arrows represent the stimulation, the solid black line segments indicate the inhibition, and the dotted lines represent the negative feedback control of the HPG axis.

Cadmium (Cd) is a toxic, nonessential transition metal and classified as a human carcinogen by the National Toxicology Program [1]. There are several sources of human exposure to Cd, including employment in primary metal industries, production of certain batteries, some electroplating processes (about 29% of year production), and consumption of tobacco products [2]. It is also of interest since the natural biogeochemical cycle of Cd has been overwhelmed. First, the concept of provisional tolerable weekly intake (PTWI) was established. The Joint FAO/WHO Expert Committee on Food Additives defines the PTWI for a chemical with no intended function as an estimate of the amount of the chemical that can be ingested weekly over a lifetime without appreciable health risk [3]. The first Cd PTWI was 400–500 *μ*g per person per week. This level was based on a critical concentration of 200 mg Cd/g kidney cortex, attainable after a dietary Cd intake of 140–260 *μ*g/d for over 50 years or 2000 mg over a lifetime. For several decades, the PTWI has been expressed more rationally in terms of the intake per kg body weight, and the value was constantly changed (Table 1). In 2010, the Consumer Product Safety Commission (CPSC) released a staff report recommending new guidance on Cd, that is, an acceptable daily intake level of 0.1 *μ*g kg^−1^ body weight per day for chronic exposure [4]. In most studies, the half-life of Cd in humans is estimated at a range of 15 to 20 years [5]. Epidemiological and experimental studies have linked the occupational Cd exposure with lung cancer and other cancers such as the prostate, renal, liver, hematopoietic system, urinary bladder, pancreatic, testis, and stomach cancers [6–8]. Exposure to Cd also severely affects the function of the nervous system [9, 10], with symptoms including headache and vertigo, olfactory dysfunction, parkinsonian-like symptoms, slowing of vasomotor functioning, peripheral neuropathy, decreased equilibrium, decreased ability to concentrate, and learning disabilities [11–13]. The neurotoxicity of Cd in children was investigated in several studies in the 1970s and 1980s but has received little attention since then. In case-control studies in which the hair concentration of Cd in a clinically defined group was compared with that of a reference group, higher concentrations of hair Cd were reported in children with mental retardation [14] and learning difficulties or dyslexia [11, 15]. In cohort studies, Thatcher et al. reported that the concentration of Cd in hair was inversely related to adjust Intelligence Quotient (IQ) [16, 17]. Other investigators [18] also reported associations between hair Cd concentrations and children's performance on visual-motor tasks. These studies clearly indicate the association of increased total Cd concentration with mental retardation and reduced visual motor abilities. Effect of Cd neurotransmitter, oxidative damage, interaction with other metals such as cobalt and zinc, estrogen-like effect, and epigenetic modification may be the underlying mechanisms (Figure 1). However, the exact mechanism(s) through which Cd elicits its neurotoxic effects is still unresolved. In this review, therefore, we focus on recent evidence from experimental and epidemiological studies, showing that Cd exposure can induce its neurotoxin effects.

## 2. The Absorption of Cd in the Nervous System and Distribution

The Cd exposure rate (based on meconium analysis) in infants was 8.5%, and the median concentration of the pollutants in the positive samples was 13.37 mg/mL. In most of these studies, the concentration of Cd in hair was measured. The CNS is especially vulnerable to damage during early neonatal development; Cd is able to readily pass to the fetus via the placenta and was detected in milk during lactation [19]. Cd can be uptaken from the nasal mucosa or olfactory pathways into the CNS; thus, the CNS is subjected to Cd toxicity [20–22]. Under normal conditions, Cd barely reaches the brain in adults due to the presence of the blood brain barrier (BBB); however, this structure is not fully developed in young animals [23]. The anatomical and physiological bases on which the choroids plexus becomes the target of xenobiotics have been examined. Cd tends to accumulate in the choroids plexus at concentrations much greater than those found in the cerebrospinal fluid (CSF) and elsewhere in brain tissues. A postmortem human study revealed that the Cd concentration in the choroids plexus was about 2-3 times higher than that found in the brain cortex [24]. As a general choroids plexus toxicant, Cd can directly damage the choroids plexus ultrastructure. Due to differences in the BBB integrity [25], Cd is thus more toxic to newborn and young rats than to adult rats. Cd can increase permeability of the BBB in rats [26] to penetrate and accumulate in the brain of developing and adult rats [27, 28], leading to brain intracellular accumulation, cellular dysfunction, and cerebral edema.

As a barrier, the choroidal epithelia are often the first and the most frequent ones to encounter metal insults from blood. Two mechanisms may aid resistance of choroids plexus to blood-borne toxicities. First, the choroids plexus contains abundant metal binding ligands that effectively sequester metal ions. Moreover, the concentration of cystine in the choroids plexus is 4-fold higher than that in the brain cortex [29]. Second, the choroids plexus owns an active defense system. The activities of superoxide dismutase and catalase are significantly higher in the choroids plexus than in cerebrum and cerebellum. Taken together, the choroids plexus likely forms the first line of defense against neurotoxicants. It must be kept in mind, however, that none of the cellular defense mechanisms would operate in an unlimited capacity. The pathophysiological changes can occur either as the threshold above which the protective capacity of the choroids plexus is exceeded or saturated, or as a direct result of barrier dysfunction. Thus, the need arises for a more comprehensive understanding of the detoxification mechanisms, such as antioxidant systems, induction of protective macromolecules (heat shock proteins, etc.), formation of specific metal inclusion bodies or binding proteins, and biotransformation reactions (methylation, conjugation, etc.) that operate in the choroids plexus.

Cd is transported along the primary olfactory neurons to their terminations in the olfactory bulbs, thereby bypassing the intact BBB. The olfactory route could therefore be a likely way to reach the brain and should be taken into account for occupational risk assessments for this metal [30–32]. Occupational inhalation of Cd can be toxic to the olfactory sense [30]. Primary olfactory neurons are regularly replaced throughout the life span. Functional recovery and regeneration of olfactory neurons has been demonstrated in laboratory animals but not confirmed in humans [33]. Intranasal exposure to Cd has been related to olfactory dysfunction in humans and to nasal epithelial damage and altered odorant-guided behavior in rodent models. The pathophysiology underlying these deficits has been partly elucidated. Optical imaging revealed significant reductions in odorant-evoked release from the olfactory nerve at a Cd chloride dose two orders of magnitude less than that required to induce morphological changes in the nerve in the same animals, demonstrating that it is a more sensitive technique for assessing the consequences of intranasal neurotoxicant exposure. This approach is potentially useful in exploring the effects of any putative neurotoxicant that can be delivered intranasal [32]. 

## 3. The Influence of Cd to Central Nervous System (CNS)

### 3.1. The Morphology Change of CNS in Response to Cd

Cd-treated embryos developed a smaller head with unclear boundaries between the brain subdivisions, particularly in the mid-hindbrain region. Embryos display normal anterior to posterior regionalization; however, the commitment of neural progenitor cells was affected by Cd [34]. Cd has been shown to produce free radicals in the brain, which may potentially damage both neurons and oligodendrocytes (OLG). OLGs are the glial cells which myelinate axons in the CNS. An early study reported that Cd toxicity affected CNS white matter [35], and one laboratory demonstrated that OLGs are direct targets of this structure [36]. Experimental studies have shown that Cd can be a potent neurotoxicant for the peripheral nervous system. Moreover, Cd has a half-life of more than 15 years in humans. Elderly workers may be more susceptible to an increased Cd body burden and may develop a peripheral polyneuropathy (PNP) over time [37]. The primary olfactory neuron is the only sensory cell directly in contact with the environment and therefore potentially exposed to airborne toxicants; so olfactory damage may represent the early action of a toxicant on the nervous system; the primary olfactory neuron may represent the early target for airborne Cd toxic action.

In cortical neurons cell culture from fetal rats at 19 days of gestation, Cd modified the neuronal morphology after a 6 h treatment in a serum-free medium with 10 *μ*M of Cd, whereas at 24 h it showed a great loss of neuronal integrity mainly evidenced by the almost complete disappearance of the axons. Lower concentrations of Cd than 1 *μ*M could induce cell apoptosis and higher concentrations of Cd than 1 *μ*M could induce necrotic cell death [9]. Cd affected the cell morphology; the morphological changes were mainly located in the neural extensions (axons and dendrites), which almost disappeared after 24 h of treatment with 1 *μ*M of Cd in a serum-free medium [9, 47]. Such morphological changes induced by Cd have also been described in other cells [48]. Cd inhibited neurite outgrowth at concentrations that decreased viability in Neuroscreen-1 cells (NS-1) models, a subclone of PC12 cells, using high content screening. The previous studies have shown that cultured oligodendrocytes are directly damaged by Cd exposure, and continuous exposure (18–48 h) of OLPs to low micromolar concentrations (0.001–25 *μ*M) of Cd significantly decreased mitochondrial metabolic activity, increased LDH leakage starting at 5 mM, and maximally activated caspase-3. These results suggest that Cd induces OLP cell death mainly by apoptosis, and at higher concentrations or with prolonged exposure to the heavy metal there is an increase in cytoplasmic membrane damage, an index of necrosis. More importantly, transient exposure to Cd is sufficient to damage OLPs and could in principle impair myelination in the neonate. Although the effects of Cd on neuronal cells in culture have been described in several papers, its effects on human central nervous system neurons *in vivo* have not yet been demonstrated. However, the recently described Cd-induced apoptosis of the motor neurons of the ventral horns in cultured explants from human fetal spinal cords (10-11 weeks gestational age) [49] suggests that the effects observed *in vitro* could actually occur also *in vivo*.

Increasing evidence has demonstrated that Cd is a possible etiological factor of neurodegenerative diseases, such as Alzheimer's disease (AD) and Parkinson's disease (PD) [50, 51]. Cerebral cortical neurons have been identified as targets of Cd-mediated toxicity and Cd-induced cell apoptosis [52, 53]. Apoptotic morphological changes induced by Cd in cerebral cortical neurons were assessed in some studies [53, 54].

*In vivo* study, the age and species of experimental animals could be effected the CNS results of damage. Perinatal exposure to Cd (50 ppm in drinking water) reduced the brain weights of pups and inhibited the activities of enzymes in nervous system, for example acetylcholine esterase, K^+^-ATPase, CNP (cyclic nucleotide phosphodiesterase), and 50-nucleotidase [55]. The Cd concentration in the choroids plexus was about 2-3 times higher than that found in the brain cortex. Cd-produced deterioration of the plexus structure can be characterized by the loss of microvilli, a rupture of the apical surface, and an increased number of blebs. Cellular debris present in the ventricular lumen may result from the breaking of the apical membrane. The epithelial cells display an abnormally high number of cytoplasmic vacuoles and lysosomes with condensed or irregular nuclei. As a general choroids plexus toxicant, Cd directly destroys the plexus ultrastructure. In both chronic (22 weeks) and acute (1–24 days) exposure models, the levels of Cd in the choroids plexus were high, while Cd in the CSF fell below the detection limit [29].

### 3.2. The Biochemical Changes of CNS in Response to Cd

The cholinergic system, with acetylcholine (ACh) as the neurotransmitter, is involved in cognitive processes, through the activation of metabotropic muscarinic and ionotropic nicotinic cholinergic receptors. The reaction responsible for the maintenance of levels of ACh is catalyzed by two cholinesterases (ChE): acetylcholinesterase (AChE) and butyrylcholinesterase (BuChE). AChE is an important biomarker for several environmental contaminants in zebrafish [56, 57]. In addition, it is also known that this enzyme plays an important role in diseases with an increasing incidence in the elderly population, such as Alzheimer disease. Zebrafish (Danio rerio) is an emergent vertebrate model for studying several biological events, such as neurochemical alterations promoted by heavy metal toxicity. This teleost possesses only the gene for AChE, which is responsible for the whole ACh degradation, being the BuChE absent. The AChE gene has already been identified, cloned, and functionally detected in the zebrafish brain. The recently reported on the effects of long-term dietary-induced exposure to Cd on the AChE activity of adult rodents' brain regions. The authors studied the changes in the activities of AChE and Na^+^, K^+^-ATPase in the cerebral cortex, hippocampus, hypothalamus, cerebellum, and striatum of adult Wistar rats, following a 5-month (long-term) exposure to an experimental diet supplemented with low levels of Cd salt or with Cd-contaminated potato tubers. The authors also assessed the behavioral (cognitive-, motor-, and anxiety-related) outcomes following the above-mentioned treatment [58]. But the other research term queried that their study can be “regarded as a significant contribution to the field, as there is paucity of information on the AChE activity in brain regions following exposure to Cd” and “the experimental protocol used in the aforementioned study [58] is exceptionally well designed in order to simulate long-term dietary-induced exposure to Cd” [59].

Cd can affect the degree and balance of excitation inhibition in synaptic neurotransmission as well as the antioxidant levels in animal brain [28]. Cd increases the serotonin sensibility in the CNS [60]. Cd inhibits the release of acetylcholine, probably by interfering with calcium metabolism [61]. A remarkable reduction in the concentration of brain galactosylceramide (30%–43%) and 3′-sulphogalactosylceramide (24%–37%) at 21, 30, and 45 days of age was observed following pre- and postnatal exposure of Cd in comparison to the respective controls. The ontogenic profile of different brain phospholipids in the Cd-exposed group showed an increase in the levels of phosphatidylethanolamine at 21 (31%), 30 (25%) and 45 (19%) days of age; the phosphatidylcholine contents increased at day 21 (14%), followed by a significant decrease at 30 (14%) and 45 (19%) days compared to age-matched controls. Brain phosphatidylinositol, phosphatidylserine, and sphingomyelin did not show any alteration at early periods of exposure but decreased significantly following continued exposure by 34%, 45%, and 21%, respectively, at 45 days of age [62].

Numerous studies have shown that cadmium caused a decrease in depolarization-evoked exocytotic release of glutamate (as well as other neurotransmitters) from nerve terminals [73]. Cd^2+^ can stimulate [^3^H]-glutamate binding in human platelets. Cd^2+^ can increase lipid peroxidation levels and reactive oxygen species (ROS) measurement in platelets. Glutamatergic system may be used as a potential biomarker for neurotoxic action of Cd in humans [74].

### 3.3. The Central Activity Changes in Response to Cd

Cd may alter the stimulus properties of morphine in adult male rat model [75]. As shown in Table 2, perinatal Cd exposure has been shown to alter behaviors and reduce learning ability. In addition, high levels of Cd and lead in children's hair were associated with learning disabilities. Motor and perceptual abilities of children exposed to Cd in uteri were significantly affected [76]. Behavioral defects, neurochemical changes, and brain lesions were reported in experimental animals, while in humans acute Cd poisoning produced Parkinsonism symptoms [50]. Blood Cd in motor neuron disease (MND, with limited disability) was higher than controls [77, 78]. Plasma Cd levels in the sporadic motor neuron disease (SMND) group were significantly increased compared to controls [78]. A recent study using a fully automated, observer-independent procedure to study morphological alterations of the central nervous system demonstrated that myalgic encephalomyelitis/chronic fatigue syndrome (ME/CFS) patients had an average decrease of the volume of the gray matter of about 8% compared to matched healthy controls [79]. The gray matter volume reduction was significantly associated with objective reduction of physical activity in ME/CFS patients. Deficits in learning and altered behaviors and activities were also observed in offspring exposed to Cd during gestational and/or lactational period. Cd induced neuronal death in cortical neurons through a combined mechanism of apoptosis and necrosis involving reactive oxygen species generation and lipid peroxidation [80]. With regard to the assessment of behavioral disorders, the cross-sectional study by Bao et al., from China, revealed a higher frequency of withdrawal, social problems, and attention problems associated with higher levels of cadmium in hair of children aged 7–16 years [71]. But another study did not find any significant association between cadmium exposure and ADHD [81].

In animal study, the role of the nanosized Cd in the causation of nervous system damages and shows the possibility of modeling human neurotoxic damage in rats [72].

## 4. The Mechanism by Which Cd Affects CNS

### 4.1. Cd and Oxidative Damage

Cd-induced injury in the cerebral microvessels is thought to be associated with oxidative stress. Following *in vivo* Cd exposure, there was an early increase followed by a later decrease in microvessel enzymes involved in cellular redox reactions, such as superoxide dismutase, glutathione peroxidase, and catalase. Thus, a depletion of microvessel antioxidant defense systems and a resultant increase in lipid peroxidation (LPO) may provoke microvessel damage [82]. Upon exposure, Cd has been shown to induce heavy metal-binding proteins such as metallothionein (MT) in various organs. MT is a low-molecular-weight protein, 6,500 Da with high cysteine content and high metal affinity, which plays a major role in the kinetics and metabolism of Cd. Four isoforms have been identified, namely, MT-I to MT-IV. Metallothionein-3 (MT-III) is specifically expressed in the brain; however, it is downregulated, and thus deficient in Alzheimer's disease [83]. The choroids plexus also expresses MT proteins. Nishimura et al. observed a strong MT immunostaining in ependymal cells and choroids plexus epithelium in younger rats (1–3 weeks old) poisoned with Cd. Thus, the sequestration of Cd by MT may partly contribute to the high accumulation of Cd in the choroids plexus [84]. Cd significantly increases the levels of lipid peroxidation in parietal cortex, striatum, and cerebellum as compared to a control group, and dexamethasone (Dx) treatment prevented the increase in LPO levels associated to Cd exposure, probably through the increase in MT content [85]. So MT as a protective mechanism against Cd-induced neurotoxicity is suggested.

In addition, mitochondria play a role in stress responses and can produce ROS when damaged. Mitochondria are indeed a major source of ROS. The enzyme COX can serve as an indicator of mitochondrial function. This is because COX dysfunction increases ROS, reduces energy stores, and impairs energy metabolism [86]. Accumulating evidences indicate that Cd-induced neuronal toxicity is due to induction of  ROS, which leads to oxidative stress [54, 87]. Recently, Cd induced ROS generation in a time- and concentration-dependent manner in PC12 and SH-SY5Y cells [54], which causes apoptosis of neuronal cells via activation of MAPKs (mitogen-activated protein kinases) and mTOR (mammalian target of rapamycin) signaling pathways [54, 87, 88].

### 4.2. Interaction and/or Impact of Other Metals Including Calcium, Zinc, and Cobalt on Cd Neurotoxicity

Cerebral cortical neurons have been identified as targets of Cd-mediated toxicity [52]. And Cd-induced cerebral cortical neurons apoptosis occurs through Ca^2+^-mitochondria signaling [52, 53]. Cd disrupts intracellular free calcium ([Ca^(2+)^] i) homeostasis, leading to apoptosis in a variety of cells including primary murine neurons. Calcium is a ubiquitous intracellular ion which acts as a signaling mediator in numerous cellular processes including cell proliferation, differentiation, and survival/death. Few studies have focused on the interaction between Cd and Ca-binding molecules, such as calmodulin.

Cd may block the influx of Ca^2+^ through membrane channels into the nerve terminal following the action potential; these decreases in calcium influx caused by Cd would be associated with an altered transmitter release [89]. Of the receptor agonists tested partially inhibited by Cd^2+^ [90]. Cd ions are often used to block high-threshold Ca^2+^ currents [91] but may also block low-threshold Ca^2+^ currents [92]. When 100 *μ*M Cd was added to the external solution, a minor fraction, 8.6% ± 8.9%, of the low-threshold current at −40 mV (holding potential −90 mV) was blocked [93]. Further studies are needed to define which molecular effects are indeed elicited by Cd-calmodulin and/or whether Cd^2+^ binds to other regulators of the Ca^2+^-induced signaling pathways.

In a new study, Cd-induced apoptosis is associated with calcium-induced massive production of ROS, dissipation of mitochondrial membrane potential (ΔΨm), cleavage of caspase-9, caspase-3, and PARP. And the results demonstrate that Cd-induced apoptosis is mediated by calcium signaling pathway, and calcium-mediated apoptosis occurs through the mitochondria-caspase signaling pathway [53].

Although Cd is not accumulated in significant quantities into the brain following exposure, it disturbs the metabolism of Cu and Zn. Because zinc (Zn) and Cd are cations of similar size and charge, and Cd has been shown to inhibit Zn uptake in a variety of systems, Cd is using transport systems that normally function to regulate Zn levels in brain. Metal analysis in the brain showed a reduction in zinc and copper levels at 15 and 21 days of age in Cd-exposed animals. It may be concluded that an early exposure of Cd may produce alteration in the development of different lipids, which may produce CNS dysfunctions with a possibility of being manifested even in later life [62]. The most well studied metallothioneins are isoforms of metallothioneins I and II that are expressed in almost all mammalian tissues. Metallothionein III is expressed in brain and is rich in zinc. Since the blood-brain barrier keeps Cd outside the CNS, reported neurotoxic effects of Cd during development are likely to be secondary to an interference of Cd with Zn-metabolism and not a direct effect of Cd on brain cells. It is therefore of importance to investigate whether neurotoxicity induced by Cd is related to mechanisms involving MT III in brain [5].

Very few articles study neurotoxin effects about the effect of cobalt (Co^2+^). This study addition of inorganic Ca^2+^ channel blockers (Cd^2+^ or Co^2+^) was used to demonstrate the Ca^2+^ dependence of  Ca^2+^-activated k^+^ currents [94]. Cd^2+^ and Co^2+^ were the nonspecific calcium channel antagonists in the study [95, 96]. Co^2+^ did not modify significantly the ATP-evoked current [97]. Application of high potassium media to growth cones inhibited neurite outgrowth, an effect that was blocked by 2 mM cobalt or 100 *μ*M Cd, suggesting that Ca^2+^ influx via voltage-gated channels contributes to glutamate-induced regulation of neuritis outgrowth [98].

### 4.3. Cd and Neurogenesis

There is evidence that relates arsenic and manganese exposure with neurodevelopmental problems in children, but there is little information on cadmium exposure. Cd-induced neurotoxicity might be caused by impaired neurogenesis, resulting in markedly reduced neuronal differentiation and axonogenesis, leading to neuronal cell death [99]. The present study compares the sensitivity of human (ReN CX) and mouse (mCNS) neuroprogenitor cell lines to chemicals using a multiplex assay for proliferation and apoptosis, endpoints that are critical for neural development. Cells were exposed to 0.001–100 *μ*M concentrations of Cd to affect proliferation and/or apoptosis. Cd decreased proliferation by at least 50% of control in either the ReN CX or mCNS cells. Compared to control, Cd decreased cell viability (ATP levels) by at least 50% in the ReN CX cells, while Cd decreased viability by at least 50% in the mCNS cells. Based on these results, BrdU is an appropriate marker for assessing chemical effects on proliferation, and human cells are more sensitive than mouse cells for this endpoint [100]. In the mammalian brain, the complex molecular pathways underlying neurogenesis provide a variety of possible targets that might be impacted by Cd exposure and identifying which pathways are disrupted is difficult.

Gender-related differences in susceptibility to chemical exposure to neurotoxicants have not received sufficient attention. There is abundant available information on the gender-specific health effects of mercury and lead, and exposure to lead seems to affect boys more than girls [101]. The internal cadmium dose is generally higher in women than in men, due to a higher gastrointestinal absorption at low iron stores. This was probably one major reason why Itai-itai disease was mainly a woman's disease. Yet, data are sparse regarding the risk for women relative to men to develop cadmium-induced kidney damage in populations exposed to low levels of cadmium [102]. Information regarding gender differences in susceptibility of cadmium is still too scarce to draw any definite conclusion. More research is highly warranted about this matter. Environmental epidemiological studies should be designed to quantify differential gender-based exposures and outcomes, and this may provide new insights into prevention strategies [101].

### 4.4. Cd and Gene Expression

Cd accumulation prior to and at birth, however, might cause irreversible or lasting changes in the brain, which in turn leads to the altered gene expression [103, 104]. The expression of several proneuronal genes including ngn1 in cell clusters, zash1a in the developing optic tectum, and zash1b in the telencephalon and tectum. Cd-treated embryos also have fewer differentiated neurons and glia in the facial sensory ganglia as indicated by decreased zn-12 expression. Also, a lower transcription level of neurogenic genes, ngn1 and neuroD, is observed in neurons [34]. Cd-induced neurotoxicity can be caused by impaired neurogenesis, resulting in markedly reduced neuronal differentiation and axonogenesis.

By contrast, caspase 3 and p53 were altered by environmental chemicals in mouse, but not in human cells. Therefore, these markers are not appropriate to assess the ability of environmental chemicals to induce apoptosis in the ReN CX cells [100]. Among the brain-specific genes, neurogranin (RC3) and myelin basic protein (MBP) are regulated by serum thyroid hormones (THs) [105]. Since Cd exposure affects THs, Cd exposure will alter expression of these genes in the brain, and furthermore, those effects might be enhanced by a hypothyroid state. In conclusion, Cd combined with MMI decreased the RC mRNA expression in the brain of female offspring. The reduced expression of RC3 mRNA may explain the effect of Cd on brain function. Perinatal Cd exposure disrupted the reproductive function of offspring indicated by the reduced expression of ERs and PgR mRNA, which was not considered to be estrogenic action.

Cd-induced apoptosis in the neuronal cells has a time- and concentration-dependent manner. Cd induces apoptosis of neuronal cells by activation of JNK, Erk1/2, and mTOR signaling network. These findings support the notion that inhibitors of these pathways may be exploited for prevention of Cd-induced Parkinson's disease, Alzheimer's disease, and other neurodegenerative disorders [88].

### 4.5. Estrogen-Like Effect (Hormones Regulating Shaft)

Cd can modify hormone levels by affecting the hypothalamic-pituitary-testicular axis in different aspects, not only via its effects on Leydig cells. Cd affected the circadian pattern release of noradrenaline, a regulator of hypothalamus hormone secretion, which resulted in changes in the daily pattern of plasma testosterone and LH levels [106]. In addition, plasma levels of pituitary hormones (e.g., LH, FSH, prolactin, ACTH) were also modified after Cd exposure [107]. Nevertheless, it remains to be investigated if Cd acts as an endocrine modulator by interacting with ERs or ARs in the testis and/or Leydig cells. The study of the hypothalamic-pituitary-gonadal (HPG) axis in animals exposed to the metal is of great interest since the levels of Cd in air, water, soil, and foods have increased by several folds in many parts of the world as a result of emissions from industrial activities. Cd accumulation increased in the hypothalamus and testes in all the Cd-treated animals, whereas the accumulation of Cd in the pituitary was found only in postpubertal rats. These data suggest that Cd exerts age-dependent effects on the hypothalamic-pituitary-testicular (HPT) axis function, and a disruption of the regulatory mechanisms of the HPG axis emerges [108]. Previous studies have shown that the heavy metal Cd mimics the effects of estradiol in estrogen-responsive breast cancer cell lines. Cd activates ER-alpha through an interaction with the hormone-binding domain of the receptor [109]. Cd globally effects HPT axis function by acting at the three levels analyzed and that an interaction between Cd exposure and age emerge [110]. The effects of Cd on sGnRH and rtERa gene expression in the brain in rainbow trout model, and these genes are strongly involved in reproduction [111]. But one study show that the effect of Cd on the ability of medaka gonads to produce steroids without the presence of physiological signals from brain, pituitary or circulating blood was examined *in vitro* [112]. Plasma levels of luteinizing hormone (LH) were not modified by Cd in both age groups, but follicle stimulating hormone (FSH) levels decreased in postpubertal rats, and was not altered in pubertal rats. Plasma levels of testosterone increased in pubertal rats but decreased in postpubertal rats [108].

In summary, it is important to note that the endocrine disruption induced by Cd is likely to be multi-factorial, mediated via its effects on Leydig cells and/or the hypothalamic-pituitary-gonadal axis.

### 4.6. Epigenetic Effect

Cd binds DNA in a weak fashion, indicating this is not a primary mode of action. Because DNA sequence is static, genetic susceptibility from DNA sequence variation cannot explain the mechanisms by which prenatal or early childhood metal exposures impact cognition and behavior later in life. Cd may well act as an epigenetic or indirectly genotoxic carcinogen since it is, in general, poorly mutagenic [6, 113]. There is growing evidence that exposure to toxicants in early life may cause later health effects. Children of women exposed to Cd during pregnancy display lower motor and perceptual abilities. High Cd body burden in children is also related to impaired intelligence and lowered school achievement. One possible mechanistic pathway for this phenomenon, which has yet to be fully explored in humans, is epigenetics. Epigenetics is the study of heritable changes in gene expression that occur without changes in DNA sequence. Such changes can have influences as profound as those exerted by mutations but, unlike mutations, are reversible and responsive to environmental influences. DNA methylation is the best studied of the epigenetic processes that regulate gene silencing. DNA methylation results in the addition of a methyl group to the 5′position of the cytosine ring in the context of CpG dinucleotides (or island) to form 5-methylcytosine (5-MeC) [114]. Several studies have demonstrated that DNA methylation was facilitated by long-term exposure to Cd [115, 116]. Oxidative stress may be a unifying process to explain these findings across different metals. Metals are known to increase reactive oxygen species production in a catalytic fashion via redox cycling. Oxidative DNA damage can interfere with the ability of methyltransferases to interact with DNA [117], thus resulting in a generalized hypomethylation of cytosine residues at CpG sites [118]. Cd-induced alterations in methylation metabolism could initiate a cascade of events including gene-specific DNA hypo- or hyper-methylation [119], resulting in aberrant gene expression and also in diminished glutathione activity leaving cells more vulnerable to oxidative stress. Although the results of these epigenetic changes on neurodevelopment have remained unexplored, given the clear importance of DNA methylation to processes of neurodevelopment, the metal-induced disruption of DNA methylation clearly deserves further study. Neurotoxicity is a common health endpoint for excess Cd exposure. Finally, there is intriguing evidence that epigenetic phenomena may underlie observed effects of fetal or early life exposure and late onset of disease. In addition, Cd inhibited DNA methyltransferases in a manner that was noncompetitive with respect to the DNA substrate. This finding is suggestive of interference in enzyme DNA interaction, possibly through an interaction of Cd with the methyltransferase DNA binding domain [115]. Failure of DNA methylation systems in the brain leads to clinical syndromes such as mental retardation and autistic-like behaviors [120]. Animal studies increasingly demonstrate that environmental factors can alter DNA methylation patterns and that these changes correlate with animal behavior [121]. Further research in Cd should include the role of epigenetics in determining long-term and late-onset health effects from metal exposure.

## 5. Summary

Cd plays a critical role in neurobiology; a growing number of clinical investigations have pointed to Cd intoxication as a possible etiological factor of neurodegenerative diseases, including Parkinson's disease, Alzheimer's disease, and Huntington's disease [50, 122, 123]. Many individuals in Europe and Asian already exceed these exposure levels, and the margin is very narrow for large groups. The question remains open about the impact of long term low level exposure to Cd on vulnerable subgroups of the general population (foetuses, pregnant women, young children, and elderly people) living in historically polluted areas where nonferrous industries are or were in operation. The neurotoxic effects of Cd were complex associated with both biochemical changes of the cell and functional changes of central nervous system, suggesting that neurotoxic effects may play a role in the systemic toxic effects of the exposure to Cd, particularly the long-term exposure. So the mechanism of Cd neurotoxicity should be enhancing, and measures should be taken to reduce cadmium exposure in the general population in order to minimize the risk of adverse health effects. The recent report on toxicity testing in the 21st century by the National Research Council of the National Academy of Sciences recommends the use of cell lines of human origin. The future of environmental research on Cd should include the role of neurotoxic in determining the long-term and late-onset health effects following Cd exposure.

## Figures and Tables

**Table 1:** Summary of the “safe” level for human contacted of Cd.

Organization/Authors	Year	Approach	Dose	Reference
FAO/WHO	1988	Intake	400–500 *μ*g/week	[3]
WHO	1992	Intake	7 *μ*g·kg^−1^ b.w./week or 1 *μ*g·kg^−1^ b.w./day	[38]
FAO/WHO	1993	Intake	7 *μ*g·kg^−1^ b.w./week	[39]
Satarug et al.	2000	Intake	30 *μ*g/day	[40]
Nasreddine and Parent-Massin	2002	Intake	10–30 *μ*g/day	[41]
WHO	2004	Drinking-water	3 *μ*g/L	[42]
FAO/WHO	2006	Food	0.4 mg/kg of the BMDL of R-Cd standard	[43]
ACGIH.	2007	Blood	5 *μ*g/L	[44]
EFSA	2009	Intake	2.5 *μ*g·kg^−1^ b.w./week	[45]
FAO/WHO	2010	Intake	5.8 *μ*g·kg^−1^ b.w./week	[46]
CPSC	2010	Intake	0.1 *μ*g·kg^−1^ b.w./day	[4]

ACGIH: American conference of governmental industrial hygienists; CPSC: Consumer Product Safety Commission; FAO: Food and Agriculture Organization; WHO: World Health Organization; b.w.: body weight.

**Table 2:** Literature review of Cd neurotoxicity in humans and rats.

Year	Study design	Age group	E/C (*n*)	Exposure to Cd	Expose pathways	Effects	Reference
1961	Cross-sectional	Male worker	106E/84C	—	Occupational exposure	Anosmia	[63]

1977	Cross- sectional	Children	31E/22C	CdH	Daily life	Neurological disorders, such as learning disabilities and hyperactivity	[11]

1981	Cross-sectional	Children	73E/44C	CdH	Daily life	Dyslexic, learning disorder	[15]

1981	Cross-sectional	Workers	49E	CdU	Occupational exposure	Polyneuropathy	[64]

1982	Cross-sectional	Children	149	CdH	Daily life	Effect on verbal I.Q.	[16]

1985	Case-control	Young men	40	CdH	Daily life	Behavioural difficulty	[65]

1985	Cross-sectional	Children	69	CdH	Daily life	Nonadaptive classroom behavior, affected behavioral development visuomotor skills ↓	[18]

1989	Cross-sectional	Male workers	31E	CdU	Occupational exposure	↓ Attention, memory, and psychomotor speed	[66]

1992	Cross-sectional	Worker	38E	CdU	Occupational exposure	90% Headache; 42% dizzy spells 21% weakness; 16% brain atrophy	[67]

1992	Cross-sectional	Worker	55E/16C	CdU	Occupational exposure	Hyposmia	[68]

1997	Case report	Old man	1	Multiple organ failure	Occupational exposure, acute	Parkinsonism	[50]

1999	Cross-sectional	Worker	13E/19C	CdU	Occupational exposure	Polyneuropathy	[37]

2000	Cross-sectional	Adult worker	42E/47C	CdU	Occupational exposure	↓ Motor speed, attention, memory ↑ equilibrium, PNP, and concentration complaints	[69]

2006	Case report	Adult workers	1	CdU	Inhale the fumes	Peripheral neuropathy	[70]

2009	Cross-sectional	Children	549	CdH	Daily life	Withdrawal, social problems and attention problems associated	[71]

2012	Wistar rats	Male	20E/20C	Intratracheal instillation	Experiment exposure	Dose- and time-dependent shift from slower to faster waves	[72]

E: exposed subjects, C: control subjects; CdU: urinary cadmium concentration; CdH: concentration of cadmium in hair; I.Q.: Intelligence Quotient.

## Abbreviations

5-MeC: 5-Methylcytosine
ACGIH: American conference of governmental industrial hygienists
Ach: Acetylcholine
AChE: Acetylcholinesterase
AD: Alzheimer's disease
ADHD: Attention deficit hyperactivity disorder
BBB: Blood brain barrier
BuChE: Butyrylcholinesterase
B.W.: Body weight
Cd: Cadmium
ChE: Cholinesterases
CFS: Chronic fatigue syndrome
CNP: Cyclic nucleotide phosphodiesterase
CPSC: Consumer product safety commission
CNS: Central nervous system
CSF: Cerebrospinal fluid
Dx: Dexamethasone
FAO: Food and Agriculture Organization
FSH: Follicle stimulating hormone
HPG: Hypothalamus-pituitary-gonadal axis
HPT: Hypothalamic-pituitary-testicular
Im: Intramuscular
IQ: Intelligence quotient
LDH: Lactate dehydrogenase
LH: Luteinizing hormone
LPO: Lipid peroxidation
MAPKs: Mitogen-activated protein kinases
mTOR: Mammalian target of rapamycin
MBP: Myelin basic protein
Mcns: Mouse cortical neural stem cells
ME: Myalgic encephalomyelitis
MND: Motor neuron disease
MT: Metallothionein
NS-1: Neuroscreen-1 cells
OLP: Oligodendrocyte progenitors
PNP: Polyneuropathy
PTWI: Provisional tolerable weekly intake
ReN CX: Human ReNcell CX
Sc: Subcutaneous
SMND: Sporadic motor neuron disease
THs: Thyroid hormones
WHO: World Health Organization
Zn: Zinc.
